# Response to Gorter et al. regarding “Acceptability and feasibility of neurocognitive assessments with adults with primary brain cancer and brain metastases: A systematic review”

**DOI:** 10.1093/nop/npad042

**Published:** 2023-08-30

**Authors:** Melissa A Carlson, Elizabeth A Fradgley, Della Yates, Sarah Morris, Jordan Tait, Christine L Paul

**Affiliations:** School of Medicine and Public Health, College of Health, Medicine, and Wellbeing, University of Newcastle, Callaghan, NSW, Australia; School of Medicine and Public Health, College of Health, Medicine, and Wellbeing, University of Newcastle, Callaghan, NSW, Australia; School of Medicine and Public Health, College of Health, Medicine, and Wellbeing, University of Newcastle, Callaghan, NSW, Australia; School of Medicine and Public Health, College of Health, Medicine, and Wellbeing, University of Newcastle, Callaghan, NSW, Australia; NSW and ACT Research and Evaluation Unit, GP Synergy, Mayfield West, NSW, Australia; School of Medicine and Public Health, College of Health, Medicine, and Wellbeing, University of Newcastle, Callaghan, NSW, Australia

We read the Letter to the Editor titled “Letter regarding ‘Acceptability and feasibility of neurocognitive assessments with adults with primary brain cancer and brain metastases: A systematic review’,” and we thank Dr M Gorter et al. for their thoughtful comments and critique of this important topic. We agree with Dr M Gorter et al. that the clinical purpose and value of the cognitive assessments are the first things to establish, and that practical issues do not warrant consideration unless the tool is found to be clinically valid. However, given there are existing reviews relating to issues of validity and clinical value of neurocognitive assessments used with adults with primary brain cancer and brain metastases,^1–4^ it is appropriate that a review be conducted to supplement that existing work by examining issues, which had been less-well explored such as the practical application and implementation of cognitive assessment.

Our definition of acceptability used in the paper^5^ is focused on successful implementation of measures and is narrower than the definition used by Dr M Gorter et al. However, our definition has been used by many others.^6–12^ We agree that the primary importance of cognitive assessment is the clinical purpose of these scales and thank Dr M Gorter et al. for their interest in the review.
